# Physiological and Psychological Effects of Medically Supervised Fasting in Young Female Adults: An Observational Study

**DOI:** 10.7759/cureus.42183

**Published:** 2023-07-20

**Authors:** Pradeep MK, Prakash B Kodali, Gulab R Tewani, Hemanshu Sharma, Aarti Nagarkar

**Affiliations:** 1 Interdisciplinary School of Health Sciences, Savitribai Phule Pune University, Pune, IND; 2 Department of Public Health and Community Medicine, Central University of Kerala, Kasaragod, IND; 3 Department of Naturopathy, Sant Hirdaram Yoga and Nature Cure Hospital, Bhopal, IND; 4 Department of Community Medicine, Sant Hirdaram Medical College of Naturopathy and Yogic Sciences for Women, Bhopal, IND

**Keywords:** calorie restriction, vitamin d, nutrition therapy, lifestyle, fasting, blood cell count

## Abstract

Background

Large-scale empirical and observational studies confirm the safety and efficacy of short-term intermittent fasting; however, prolonged fasting (fasting for more than two days or more) is not well studied. This study investigated the safety, physiological, and psychological effects of a medically supervised fasting (MSF) regimen on healthy volunteers.

Methods

In this observational study, 117 female participants with an average age of 21.02 (± 1.45) years underwent 10 days of medically supervised fasting. Daily symptom scores and 24-hour food recalls were collected, along with serum levels of vitamin D and calcium, a complete blood count, anthropometric measurements, quality of life (QoL), and subjective mood, anxiety, and depression scores, at the baseline and at the end of fasting.

Results

Compared to baseline, significant improvements were observed in vitamin D levels (z = -8.79, p = 0.000), calcium levels (z = -4.08, p = 0.000), red blood cell count (z = -4.61, p = 0.000), and hemoglobin levels (z = -5.57, p = 0.00). Improvements were observed in physical QOL (t (116) = -4.51, p = 0.000); psychological QOL (t (116) = -4.70, p = 0.000); and social QOL (t (116) = -2.68, p = 0.008). We also observed significant reductions in body weight (55.83 (±11.38), 52.99 (±10.94); p = 0.00) and other anthropometric measures. More than 80% (n = 94) experienced at least one symptom associated with fasting. The symptoms associated with fasting changed significantly (p<0.05), with most symptoms peaking from day 3 to day 7 of fasting with remission from day 8 onwards.

Conclusion

Our findings suggest that MSF is associated with significant improvements in physiological and psychological variables. The symptoms associated with MSF are to be considered in clinical decision-making, and follow-up of patients on fasting therapy, particularly during the peaking of symptoms, may be warranted.

## Introduction

Numerous clinical trials, reviews, and preclinical studies on fasting in the recent decade have strengthened the recommendation of fasting from a traditional and cultural practice to an evidence-based therapeutic practice [1-3]. Barring the studies on religious fasting (Ramadan fasting, Daniel fasting) [4] and a few other clinical studies, most of the studies on fasting are on short-term intermittent fasting (16-48 hours) and calorie restriction [5].

Prolonged fasting (fasting lasting more than two days up to 21 days or more) is reported to be beneficial in treating various clinical conditions by attenuating body weight and improving cardiometabolic markers [6,7]. Furthermore, Tewani et al. and Arankalle et al. reported the impact of prolonged medically supervised fasting on the biochemical parameters of healthy volunteers and diseased populations, respectively. They found 10-day medically supervised fasting to increase vitamin D levels and hemoglobin levels, reduce inflammation, reduce body weight, and improve the quality of life [8, 9].

A recent clinical trial comparing prolonged fasting and short-term fasting among male healthy volunteers reported prolonged fasting for six days to enhance insulin release and glucose tolerance capacity [10]. Grundler et al. have shown prolonged fasting for 14 days to significantly improve lipoprotein levels and their sub-classes in a group of forty middle-aged adults [11]. Besides these studies reported above, we are not aware of any large-scale studies on medically supervised prolonged fasting in healthy adults that have established its safety and efficacy as of 2023.

Furthermore, except for a single study from Arankalle et al. [9], the rest of the studies of prolonged fasting were either on diseased populations or older adults. Understanding the impact of prolonged fasting on the physiological and psychological variables of young, healthy participants may shed light on its safety and potential utility in a variety of clinical conditions. Moreover, documenting the safety of medically supervised fasting (MSF) with respect to known fasting-associated symptoms (such as headache, fever, fatigue, gastric issues, sleep disturbances, and giddiness) could facilitate clinical decision-making and protocol development.

The aim of the present study was to assess prospectively for the first time the impact of Medically Supervised Fasting (MSF-supervised periodic fasting for ten days) on selected physiological and psychological parameters among young females. The study also seeks to document the onset and changes in fasting-associated side effects among young females undergoing medically supervised fasting.

## Materials and methods

Study settings and ethics

The present observational study was carried out at a private yoga and naturopathy medical college in India. The study was approved by the Institutional Ethics Committee, vide F. No. 12/SHMCNYS/IEC/P28. All the study participants signed an informed consent form, expressing their willingness to share the data with the investigators.

Study design

This single-arm observational cohort study focused on the changes in biochemical, psychological, and safety parameters associated with exposure to MSF (medically supervised fasting). The investigators did not participate in the diet planning or influence the food choices of the participants.

Participants

The participants were a group of female medical students aged between 18 and 25 who practiced ten days of medically supervised fasting as a part of their medical education program (Bachelors in Naturopathy and Yogic Sciences). The participants were recruited using a quota-sampling approach.

We excluded people from participating in this study if they are underweight (Body Mass Index (BMI) <18.5), overweight (BMI >25 to <30), or obese (BMI ≥ 30); have a known nutritional deficiency; have any co-morbid conditions like cardiovascular disease, diabetes mellitus, musculoskeletal disorders, or skin disorders; are on any supplementation programs; are taking any medications; or have any history of psychiatric illness.

Sample size

Considering the study’s objective, a quota sampling approach was employed to sample the participants for the study groups. The study included a sample of 118 participants who fasted based on their pre-designed academic allocation between September 2021 and September 2022.

The sample size was estimated based on the anticipated values of vitamin D levels among subjects in a MSF group in a previous study [8], at a 95% confidence level, 95% power, and an alpha of 0.05 [12]. We further adjusted the sample size by employing a design effect of 3.0 for quota sampling [13] and a loss to follow-up of 30%, resulting in a sample size of 118. We also conducted a post-hoc power analysis [14], which yielded that the study sample has significant power (>0.80).

Exposure

The participants underwent a 10-day MSF, which included eight days of fasting (total calorie intake per day ≤ 500 Kcal), one day of the preparatory phase, and one day of the refeeding phase (total calorie intake per day ≤ 1000 Kcal) [6]. All MSF participants resided at the same facility and were closely monitored by their professors, who are licensed yoga and naturopathy physicians. Furthermore, the entire MSF program was overseen by three investigators stationed at the research site during every phase of the study. During the fasting period, all the participants had freshly squeezed puree or concentrated fruit and vegetable juices, soups, and/or lemon honey juice. During the preparatory and re-feeding phases, the participants consumed khichidi (a South Asian cuisine), boiled vegetables (bottle gourd), raw vegetable salads/sprouts, and mixed vegetable soups. The consumption pattern and portion size varied from person to person. The MSF is described in detail in the Appendices.

Outcome measures

The following outcome variables were measured at baseline (before the initiation of fasting) and at the end of the fasting day (on day 10).

Primary Outcome Measure

Serum vitamin D: The changes in the serum vitamin D levels of the fasting individuals were measured using a fully automated chemiluminescent immunoassay.

Secondary Outcome Measures

Serum total calcium: The changes in the serum total calcium levels of the fasting individuals were measured using the CALC-Arsenazo III Method.

Complete blood count: Measurement of the complete blood count was performed using a fully automated bidirectional analyzer (6-Part Differential SYSMEX XN-1000TM). The variables included under the complete blood count were neutrophils, lymphocytes, monocytes, eosinophils, basophils, total red blood cell counts (RBC), hemoglobin, hematocrit, mean corpuscular volume (MCV), mean corpuscular hemoglobin concentration (MCHC), mean corpuscular hemoglobin (MCH), RBC distribution width (RDW), platelet distribution width (PDW), mean platelet volume (MPV), and platelets.

Anthropometric measures: Height, weight, body mass index (BMI), waist circumference, and hip circumference were measured in both cohorts using standard protocols [15].

Vital scores: Basic vital signs such as blood pressure (sitting position), pulse rate, and respiratory rate were measured in all fasting participants.

Visual analogue scale (VAS) scores for health, mood, anxiety, and depression: VAS consists of a continuous horizontal line of 10 cm [16]. The scale ranged from zero to ten, where ‘zero’ represents the least extreme and ‘ten’ represents the most extreme. A higher score on the health VAS was considered an enhanced health status, whereas a higher score on the mood, anxiety, and depression VAS was considered the worst mental health status.

World Health Organization Quality of Life (WHO-QoL BREF) [17]: A 26-item quality of life (QoL) instrument in English that measures QoL in four domains, namely physical, psychological, social, and environmental, was used to assess the QoL of the participants.

Symptom scores: The self-reported symptoms during the fasting period were collected in both cohorts using a dichotomous symptom marker ("Yes" or "No"). The participants were asked to mark their symptoms like headache, fever, body ache, fatigue, hyperacidity, sleep disturbances, diarrhea, constipation, giddiness, and vomiting on a sheet provided to them before the initiation of fasting.

Food recall diary: A 24-hour food recall has been collected from the MSF participants once during the fasting days to assess the total calorie and macronutrient intakes. The participants were requested to tabulate the quantity of food items consumed using predetermined types of utensils (Spoons, Bowels, and glasses) or in grams.

Data analysis

The data entry and cleaning were done using Microsoft Excel 2019 (Microsoft® Corp., Redmond, WA, USA), and the data analysis was conducted using the Statistical Package for Social Sciences (SPSS) (IBM Corp., Armonk, NY, USA). A case deletion was undertaken to address missing data. The baseline and post-fasting measures for study parameters (i.e., serum Vitamin D, serum total calcium, complete blood count, anthropometric measurements, and WHO quality of life) were compared.

Specifically, a paired t-test was used to compare the changes in study parameters from baseline to post-fasting. Prior to the analysis, the assumptions of the paired test were assessed and adhered to. The normality of the variables was assessed using the Shapiro-Wilk test for normality [18]. For the parameters that were non-normal, the Wilcoxon Signed Ranked Test [19] was used to compare the baseline and post-fasting measures. Glass’s delta was computed as a measure of effect size, and the significance level (α) was set as a p-value of ≤ 0.05.

The onset of symptoms such as headache, fever, constipation, giddiness, etc. were analyzed to provide a descriptive account of the trend in the development and remission of the symptoms. The rate of symptom onset per 100 person-days was calculated. Cochrane Q was computed as a measure to test for the significance of the change in the onset of symptoms from day 1 to day 10 of the medically supervised fasting.

## Results

Among the 118 participants selected, 117 completed the 10-day medically supervised fast. One participant dropped out on the seventh day of fasting due to an adverse event and hospitalization with severe diarrhea, fever, and urine retention. The average age of the study participants was 21.02 (± 1.45) years, and they consumed on average 275.21 (±65.85) Kcal during the fasting days. Furthermore, the participants consumed on average 62.5 (±17.23) grams of carbohydrates, 4.01 (±1.81) grams of proteins and 1.07 (±0.44) grams of fat during the fasting day.

Impact of MSF on Vitamin D and serum total calcium levels

We observed a statistically significant change in the Vitamin D levels from the pre-fasting baseline (11.92±7.73) to post-fasting at the end of the 10-day MSF program (16.74±8.09) (z= -8.79, p=0.000). A similar significant improvement was observed in serum total calcium levels (see Table 1).

**Table 1:** Changes in study parameters from baseline to the end of the 10-day medically supervised fasting RBC - Red Blood Cells; PCV - Packed-cell volume; MCV - Mean corpuscular volume; MCH - Mean Corpuscular Haemoglobin; MCHC - Mean Corpuscular Haemoglobin Concentration; RDW SD - Red blood cell distribution width Standard Deviation; PDW - Platelet distribution width; MPV - Mean platelet volume; Effect Size: Glass delta was used as a measure of effect size. The significance of the differences in study variables at baseline and post Medically Supervised was tested using paired t-test (for normally distributed variables) and Wilcoxon Signed Ranked Test (for non-normal variables).

Variables (units) (reference range)	Baseline	Post Fasting	Effect Size	p-value
	Mean (±SD)	Mean (±SD)		
Vitamin D (ng/ml) (30-100 ng/ml)	11.92 (±7.73)	16.74 (±8.99)	0.62	0.000
Calcium (mg/dl) (8.8-10.6 mg/dl)	9.54 (±0.45)	9.84 (±1.4)	0.67	0.000
Neutrophils (%) (40-80%)	57.24 (±0.8.49)	52.12 (±1.78)	-0.60	0.000
Lymphocytes (%) (20-40%)	34.93 (±8.13)	39.29 (±10.23)	0.54	0.000
Monocytes (%) (0-10%)	4.77 (±1.49)	5.57 (±1.93)	0.54	0.000
Eosinophils (%) (0-6%)	2.53 (±2.11)	2.71 (±2.26)	0.09	0.009
Total RBC (x 10^6^/µL) (3.948 x 10^6^/µL)	4.56 (±1.17)	4.72 (±0.3)	0.14	0.000
Hemoglobin (g/dL) (12-15 g/dL)	12.84 (±3.31)	13.29 (±2.93)	0.14	0.000
Hematocrit PCV (%) (36-46%)	41.92 (±6.65)	42.16 (±5.71)	0.04	0.918
MCV (fL) (83-101 fL)	92.24 (±12.15)	90.37 (±10.58)	-0.15	0.000
MCH (pq) (27-32 pq)	29.59 (±7.6)	29.16 (±3.16)	-0.06	0.000
MCHC (g/dL) (31.5-34.5 g/dL)	30.78 (±2.24)	31.53 (±2.27)	0.33	0.000
RDW SD (fL) (39-46 fL)	50.00 (±8.66)	47.28 (±8.45)	-0.31	0.000
PDW (fL) (9.6-15.2 fL)	13.71 (±3.64)	14.99 (±4.09)	0.35	0.000
MPV (fL) (6.5-12 fL)	11.26 (±1.44)	11.75 (±1.39)	0.34	0.000
Platelet (10^3^/µL) (150-400 x 10^3^/µL)	301.41 (±8.37)	292.41 (±70.6)	-0.11	0.008

Impact of MSF on complete blood count

The complete blood count of the participants revealed significant changes in the blood picture at the end of fasting. With respect to the white blood cells (WBC), we found the MSF to be associated with a significant reduction in neutrophil percentage (from 57.24% to 52.12%) and an increase in lymphocyte levels post-fasting (34.93% to 39.29%). Similarly, MSF was associated with a significant increase in monocytes and eosinophils (see Table 1).

A mild yet significant increase was observed in the total RBC count (from 4.56 to 4.72), hemoglobin (from 12.84 to 13.29), MCHC, PDW, and MPV at the end of the fasting period (see Table 1).

Furthermore, we assessed the clinical significance of the findings by categorizing the parameters into deficiency, low levels, and high levels based on the standard biological reference range. A Cochrane Q test revealed significant changes in neutrophils, hemoglobin, MCV, MCHC, and RDW SD levels (see Table 2).

**Table 2:** Clinical significance of the medically supervised fasting with reference to the study parameters #Deficiency/Low refers to the values of the parameters assessed lesser than the biological reference range; *Higher values at baseline than the biological reference range PCV - Packed-cell volume; MCV - Mean corpuscular volume; MCH - Mean Corpuscular Haemoglobin; MCHC - Mean Corpuscular Haemoglobin Concentration; RDW SD - Red blood cell distribution width standard deviation; PDW - Platelet distribution width

Study Parameters	Baseline (n)	Post Fasting (n)	Cochrane Q	P value
Vitamin D Deficiency#	113	111	1.00	0.317
Calcium Deficiency	5	4	1.00	0.317
Low neutrophils	1	19	18.00	0.000
Low lymphocytes	6	5	0.14	0.705
Monocytes*	1	0	1.00	0.317
Eosinophils *	7	8	0.14	0.705
#Low hemoglobin	32	19	9.94	0.002
Low Hematocrit PCV	8	7	0.14	0.705
Low MCV (fL)	23	30	5.44	0.020
Low MCH (pq)	41	40	0.33	0.564
Low MCHC (g/dL)	82	43	39.00	0.000
Low RDW SD (fL)	5	10	5.00	0.025
Low PDW (fL)	4	2	1.00	0.317
Low Platelet (10^3^/µL)	5	7	0.33	0.564

Impact of MSF on anthropometric measures

We noted that the medically supervised fasting resulted in a significant reduction in weight, waist circumference, hip circumference, and body mass index towards the end of the fasting regimen. The MSF group had an average reduction of 2.84 kilograms (6.26 pounds) from baseline to the conclusion of fasting (see Table 3). While no significant changes were observed in the blood pressure, marginal changes were noted in the pulse rate and respiratory rate.

**Table 3:** Impact of medically supervised fasting on anthropometric measures, vitals and quality of life SBP - Systolic Blood Pressure; DBP - Diastolic Blood Pressure; VAS - Visual Analog Scale; QOL - Quality of Life; RR - Respiratory Rate; PR - Pulse Rate, Effect Size: Glass delta was used as a measure of effect size. The significance of the differences in baseline and post Medically Supervised fasting was tested using paired t-test (for normally distributed variables) and Wilcoxon Signed Ranked Test (for non-normal variables).

Variables	Baseline	Post Fasting	Effect Size	P value
	Mean (±SD)	Mean (±SD)		
Weight (kg)	55.83 (±11.38)	52.99 (±10.94)	-0.25	0.000
Waist (inches)	32.72 (±3.89)	31.38 (±3.77)	-0.34	0.000
Hip (inches)	37.77 (±3.85)	36.48 (±3.62)	-0.33	0.000
Body Mass Index (kg/m^2^)	22.48 (±4.06)	21.33 (±3.91)	-0.28	0.000
SBP (mm Hg)	107.36 (±7.52)	108.87 (±7.66)	0.20	0.106
DBP (mm Hg)	74.84 (±6.86)	75.30 (±6.49)	0.07	0.428
PR (per min)	77.00 (±8.87)	72.07 (±9.05)	-0.56	0.000
RR (per min)	17.91 (±4.77)	18.90 (±4.47)	0.21	0.060
VAS-Health	6.56 (±1.66)	6.85 (±1.81)	0.17	0.040
VAS-Mood	5.95 (±1.85)	6.27 (±11.38)	0.17	0.062
VAS-Anxiety	4.03 (±2.15)	3.38 (±2.2)	-0.30	0.000
VAS-Depression	3.62 (±2.48)	3.03 (±2.35)	-0.24	0.018
QoL-Physical	62.04 (±16.14)	69.50 (±13.6)	0.46	0.000
QoL-Psychological	60.32 (±15.05)	67.22 (±14.36)	0.46	0.000
QoL-Social	66.66 (±14.74)	68.56 (±15.32)	0.13	0.147
QoL-Environmental	62.86 (±14.56)	65.95 (±14.81)	0.21	0.008

Impact of MSF on perceived health, mood, anxiety, and depression

A marginal improvement in perceived health towards the end of fasting was observed among the participants who completed the fasting regimen. We also noted a statistically significant reduction in anxiety (captured through a visual analog scale). The anxiety score decreased from 4.03±2.15 at baseline to 3.38±2.2 by the end of the fasting period. A similar significant reduction was found for depression (see Table 3).

Impact of MSF on quality of life

We found significant improvements in at least three domains of quality of life among the participants. The physical QOL from baseline (62.04±16.14) to the end of the fasting regimen (69.50±13.60) was documented to have a significant increase (t(116) = -4.51, p = 0.000). Similarly, psychological QOL and environmental QOL were found to have significant improvements (see Table 3).

Symptoms associated with MSF

Except for one participant who was lost to follow-up due to hospitalization, no serious adverse events were observed in the participants. 80.3% (n = 94) of the participants experienced at least one of the ten symptoms associated with fasting, including headache, fever, fatigue, body ache, sleep disturbances, diarrhea, constipation, hyperacidity, giddiness, and vomiting.

Headache was the most prominent symptom, with a rate of occurrence of 14.2 (12.3-16.3) cases per 100 person-days of fasting regimen, followed by fatigue 13.2 (11.4-15.3), body ache 10.3 (8.7-1.2), giddiness 7.0 (5.7-8.6), and constipation 6.8 (5.5-8.3) (see Table 4).

**Table 4:** Fasting-associated symptoms among the study participants (n=117) df=degrees of freedom; CI=Confidence Interval

Symptoms	Cases per 100 person days (95% CI)	Cochrane Q (df=9)	p-value
Headache	14.2 (12.3-16.3)	101.309	0.000
Fever	4.9 (3.8-6.3)	25.442	0.003
Fatigue	13.2 (11.4-15.3)	76.121	0.000
Hyper Acidity	1.3 (0.8-2.1)	9.000	0.437
Body ache	10.3 (8.7-1.2)	42.267	0.000
Sleep Disturbances	6.0 (4.7-7.5)	45.652	0.000
Diarrhea	1.4 (0.8-2.2)	20.286	0.016
Constipation	6.8 (5.5-8.3)	11.868	0.221
Giddiness	7.0 (5.7-8.6)	39.679	0.000
Vomiting	2.6 (1.8-3.7)	21.289	0.011

**Figure 1:**
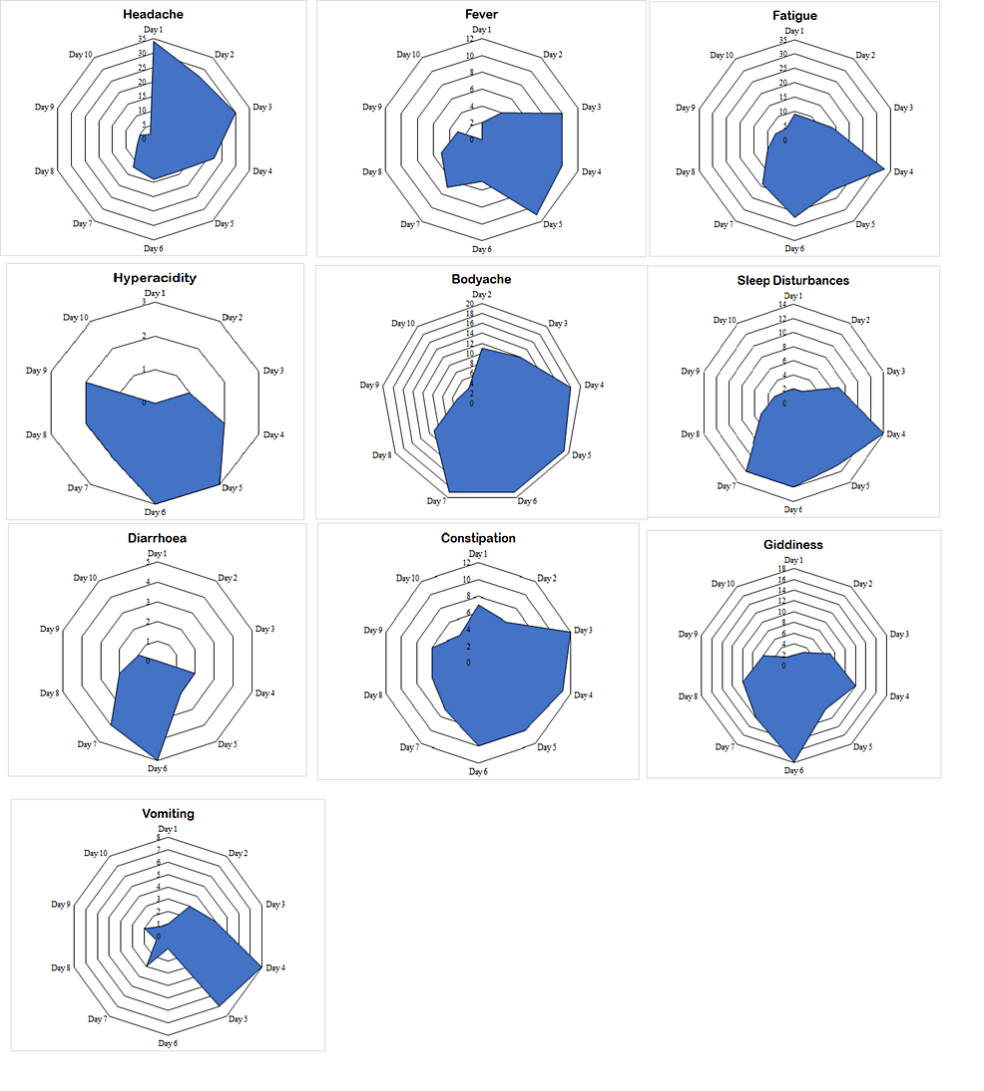
Onset and changes in fasting-associated symptoms among participants undergoing medically supervised fasting.

The onset and intensity of symptoms were found to have a pattern, with headaches being the most prominent symptom in the initial three days of fasting. The symptoms of fever, fatigue, body ache, constipation, and sleep disturbances peak from days three to seven of the fasting regimen. The remission of symptoms was observed from day seven onward (see Figure 1). The changes in the symptoms were observed to be significant based on a Cochran’s Q test (see Table 4).

## Discussion

The data from the present study demonstrate the safety, physiological, and psychological changes associated with MSF in a large sample of healthy volunteers. The findings suggest that MSF may have potential health implications; however, monitoring the symptoms associated with MSF may be considered in clinical decision-making.

We observed a significant increase in the vitamin D levels of the MSF group post-fasting. Earlier research also suggested that fasting under medical supervision could improve vitamin D levels in both healthy volunteers and patients with chronic diseases [8, 9]. The present finding substantiates the earlier reports on fasting and vitamin D levels. An earlier report reported that a hypocaloric diet, which can induce 10% weight reduction, can independently raise vitamin D levels by 34% without any supplementation [20].

The changes in the vitamin D levels of the present cohort may be due to the significant weight loss (2.84 kg) associated with MSF. The other possible mechanisms by which MSF may have had an influence on vitamin D levels may be due to the impact of fasting on: (i) parathyroid hormones [21], (ii) adipose tissue metabolism [22], and (iii) modulating the gut microbial dysbiosis [23]. Nonetheless, the findings of the MSF group are encouraging to impart MSF as a measure to mitigate vitamin D deficiency or insufficiency, which is considered a public health issue that requires immediate attention [24]. Meanwhile, the long-term impact of fasting on serum vitamin D levels needs to be explored to understand the sustainability of the present recommendations.

We observed an increase in serum calcium in the MSF group after fasting. It is well known that in the presence of vitamin D, calcium is actively absorbed from the small intestine [25]; therefore, increased vitamin D levels in the MSF group may be the reason for the increased serum calcium levels in the MSF group. The findings implicate the potential role that MSF can play in augmenting bone health. However, it is to be noted that the estimation of corrected calcium levels with account for albumin levels in the body will be a more appropriate method to assess the calcium levels.

MSF has been associated with a significant effect on almost all blood cells, as measured by a complete blood count. Neutrophils, which are thought to be the first line of defense for the innate immune system and an integral component of the inflammatory pathway [26], were found to be reduced after fasting. Similarly, we found a significant rise in the lymphocyte, monocyte, and eosinophil counts after MSF. Our finding supports the earlier report from Koscielniak et al., who have shown that neutrophil and eosinophil counts increase during the feeding phase compared to the fasting phase [27].

The increase in monocyte counts in the MSF group is contrary to earlier reports on short fasting, which have shown short fasting reduces monocyte counts [28]. However, the present study differs from the previous study with respect to the duration of fasting and nutrient intake. The current findings substantiate the earlier reports on the impact of fasting on immune cells, which state that different types of fasting induce varying responses in the immune cells, which may be similar or opposite [29]. Interestingly, the changes in the neutrophils, leucocytes, monocytes, and eosinophils are well within the normal physiological range, which suggests the role of fasting as a potential regulator of immunity and inflammation.

We observed a significant increase in hemoglobin levels and RBC, which may be due to the protective and rejuvenating effects of prolonged fasting on the hematopoietic stem cells [30]. A recent study on seven days of water-only fasting also reported an increase in RBC levels and hemoglobin. They also reported that the increase in RBC levels is independent of RBC apoptosis, suggesting an upregulation of hematopoietic systems [31].

Our findings differ from two of our previous studies on fasting, where we found no significant change in the study participants' hemoglobin levels [8, 9]. However, those studies are limited by sample size issues or patient heterogeneity. Nevertheless, our inferences indicate that fasting may have beneficial effects on RBC levels and hemoglobin. Further, the other changes observed in the RBC-related indices like MCHC, PDW, and MPV and the reductions in MCH, MCV, and RDW were within the normal range. This may be considered the residual effect of the changes in hemoglobin and RBC levels [32].

We observed a similar normative reduction in the platelet counts of the study participants post-fasting. Earlier studies on MSF also reported a reduction in platelet levels post-fasting [9,33]. While the exact reason for these changes in blood cells was not explored in this study, these findings reiterate the homoeostatic potential of fasting and warrant its inclusion as a therapy in an array of clinical conditions. Aside from statistical significance, we found clinically meaningful changes in a few biochemical indicators, which support MSF's clinical value.

MSF did not significantly impact the vitals except for the reduction in the pulse rate. We further observed significant reductions in weight, BMI, waist circumference, and hip circumference. The present finding adds to the growing body of literature that suggests the beneficial effects of fasting on body composition, weight, and other obesity-related profiles [34].

MSF had a significant impact on psychological well-being by reducing anxiety and depression. These results are consistent with earlier research on fasting, which has demonstrated that it lowers anxiety and depressive symptoms [35]. Fasting has also been linked to an increase in mood swings and irritability due to changes in dietary practices and meal timings [36, 37]. Although not significant, our participants reported increased mood swings after fasting. Future MSF programs should consider including activities that can prevent mood disturbances or irritability during fasting. Further, the participants reported an improvement in their self-reported health status along with an improvement in their physical, psychological, and environmental QoL. The current findings are consistent with a prior investigation of fasting that revealed its long-term effects on quality of life [38].

This study also included data on the MSF's safety in terms of symptoms experienced. The MSF was associated with an incidence of headache, fever, fatigue, body ache, sleep disturbance, diarrhoea, constipation, hyperacidity, and vomiting. However, these symptoms are reported to be a common reaction to fasting, as per the international expert consensus on fasting [6]. An earlier study evaluating the safety of fasting also reported headache, sleep disturbance, hunger, and muscle pain as the main symptoms observed during fasting [5].

Our study has found additional symptoms like fever, fatigue, diarrhoea, constipation, and vomiting associated with fasting. The increase in symptoms among the MSF group may be due to the extreme calorie restriction followed by the MSF group participants. We could observe a clear trend in symptoms among the MSF participants towards the end of fasting. This trend of symptoms observed in MSF may have significant implications in clinical practice, as a thorough knowledge of expected symptoms will be helpful in framing guidelines for MSF in patients with various clinical conditions.

This is the first study to present the impact of MSF on selected biochemical, anthropometric, and safety profiles in a larger sample size with identical characteristics. There are some limitations related to the present study. Due to a long observation period, several confounding factors like daily routine and physical activity may affect the outcomes, which should be considered limitations. The use of only a 24-hour food recall in the MSF group for calculating the calories may also be considered a limitation. Dietary portion size differences between participants may also be regarded as a limitation. Furthermore, due to operational reasons, the MSF groups underwent fasting in different batches throughout the year. The varying environmental conditions during the different seasons may have an influence on the outcome variables; hence, this could be considered a potential confounder. Additionally, the present study included only female participants due to the non-availability of male participants in the study setting, which limited the MSF group’s findings to women.

## Conclusions

MSF appears to be safe and is significantly associated with improvements in serum vitamin D, serum calcium, and anthropometric measures; however, the role of variable genetic, dietary, and lifestyle behaviors in modifying these outcome measures must be investigated to substantiate these associations. The findings from the present study provide insights on the points to be considered while designing fasting programs, which need to be validated by well-designed randomized control trials.

## Appendices

**Table 5:** Summary of food items consumed by the medically supervised fasting group *The food items mentioned are based on the collective 24-hour food recall. There are variations in individual food choices, fasting; MSF - Medically supervised fasting

Meal Timings	MSF Group Preparatory/Re-feeding days diet (1000 Kcal approximately)	MSF Group Fasting days diet* (500 Kcal approximately)	Meal Timings
Morning 8 am	Lemon honey water	Lemon honey water, Coconut water	Early morning (4 am-6 am)
Mid-morning 11 am	Green gram sprouts Fruit bowl (Apple, Banana, Mango, Papaya), Dates	Green juice, Carrot beet juice, Lemon honey water	
Lunch 1 pm	Mixed vegetable khichdi, Vegetables (Peas, bottle gourd) Salads (Cucumber, carrot, beet)	Papaya juice, Carrot juice, Apple juice, Watermelon juice, Lemon honey water	
Evening 4 pm	Mixed vegetable soup	Orange juice, Lemon honey juice, Jaggery water	Late evening (7 pm onwards)
Dinner 7 pm	Vegetable Khichdi, Vegetable Daliya Chutney (coconut, Coriander, Black gram)	Lemon honey water, Tomato soup, Bottle gourd soup, Carrot beet tomato soups, Mixed vegetable soup	
